# Clinical Outcomes and Complications of Preoperative Embolization for Intracranial Giant Meningioma Tumorectomy: A Retrospective, Observational, Matched Cohort Study

**DOI:** 10.3389/fonc.2022.852327

**Published:** 2022-03-08

**Authors:** Yi Yin, Yuhong Li, Zhouyang Jiang, Chao Zhang, Hongfei Ge, Zhi Chen, Rong Hu, Yujie Chen, Xuegang Li, Fei Li, Hua Feng

**Affiliations:** ^1^ Department of Neurosurgery, Southwest Hospital, Third Military Medical University (Army Medical University), Chongqing, China; ^2^ Chongqing Clinical Research Center for Neurosurgery, Southwest Hospital, Third Military Medical University (Army Medical University), Chongqing, China; ^3^ Chongqing Key Laboratory of Precision Neuromedicine and Neuroregenaration, Southwest Hospital, Third Military Medical University (Army Medical University), Chongqing, China

**Keywords:** meningioma, embolization, outcome, complication, preoperative

## Abstract

**Objective:**

The potential benefits of preoperative embolization for intracranial meningiomas are still under debate. We aimed to investigate whether preoperative embolization can improve surgical and functional outcomes, based on controlling patient- and tumor-related confounding factors.

**Methods:**

We reviewed all meningioma cases in our department from January 2016 to May 2021. Cases in the nonembolization cohort were matched to the embolization cohort by 1:1 ratio propensity score matching, through controlling patient- and tumor-related confounds. Surgical outcomes, complications, and functional outcomes were retrospectively compared between these two groups.

**Results:**

Sixty-six cases in each group were included in our study after being matched. We did not find any significant differences of estimated blood loss (600.00 (400) vs. 500.00 (500.00) ml, *p* = 0.31), decrease of HGB level (30.81 ± 15.82 vs. 26.59 ± 12.90 g/L, *p* = 0.09), gross total resection rate (74.24% vs. 77.27%, *p* = 0.68), surgical time (302.50 (136) vs. 300.00 (72) min, *p* = 0.48), blood transfusion rates (53.03% vs. 42.42%, *p* = 0.35), blood transfusion volume [650.00 (657.50) vs. 535.00 (875.00) ml, *p* = 0.63] between the embolization group and nonembolization group. The number of patients who experience postsurgery complications were significantly higher in the nonembolization group (39.39% vs. 21.21%, *p* = 0.02). Patients in the nonembolization group were more likely to have a higher rate of mRS decline postsurgery (31.82% vs. 15.15%, *p* = 0.04).

**Conclusion:**

Our study showed significant lower rates of surgical complications and long-term disabilities of meningioma patients treated with preoperative embolization. There were no significant differences in estimated blood loss, surgical time, and blood transfusion volume between embolization and nonembolization groups.

## Introduction

Meningiomas are the most common type of primary brain tumors, accounting for one-third of all central nervous system (CNS) tumors (1). Meningiomas always tend to be rich in vascularity, which complicates surgical resection due to substantial intraoperative blood loss. Devascularization of meningioma by preoperative endovascular embolization of feeding vessels was firstly introduced by Manelfe et al. (2). The intentions of such adjunctive therapy are to reduce surgical blood loss, soften tumor, and shorten the surgical time (3–6). However, discrepancies are found between results reported by different groups (7–9). Recent updated meta-analysis study indicates no clear benefit is observed in operative and postoperative outcomes of embolization (10), which is inconsistent with findings in an earlier meta-analysis (11).

Currently, no consensus or guidelines have elucidated the issue whether preoperative embolization benefits patients with meningiomas. Surgeons tend to embolize large and highly vascularized meningiomas, which may contribute to intergroup selection bias of baseline characteristics. Thus, moderate to high heterogeneity is observed in meta-analysis (10). An investigation performed by Przybylowski et al. has found that the surgical outcomes exhibit no obvious improvements using cohort matching method to control patient- and tumor-related confounds. As only WHO grade I meningiomas, which are generally less aggressive and complicated than WHO grades II and III meningiomas, are enrolled in this study, that limits generalizability of interpretation. Furthermore, their results demonstrate that embolization is found to lead to a greater chance of clinical improvement (12).

Therefore, this cohort-matching study retrospectively was performed with reviewing data of patients with meningioma who underwent with/without preoperative embolization at the Department of Neurosurgery of the Southwest Hospital Affiliated to Army Medical University from January 2016 to May 2021. Cases in the nonembolization cohort were matched to the embolization cohort by a 1:1 ratio propensity score matching through controlling patient- and tumor-related confounds. Surgical outcomes, complications, and functional outcomes were retrospectively compared between these two cohorts. The aim of the study is to validate the effect of preoperative embolization on surgical outcomes, complications, and functional outcomes in patients with meningioma.

## Materials and Methods

### Patients

Patients diagnosed with meningioma were enrolled in the present study at the Department of Neurosurgery of the Southwest Hospital Affiliated to Army Medical University from January 2016 to May 2021. All eligible patients with or without preoperative superselective tumor embolization were recruited in this study. The inclusion criteria included supratentorial WHO grades I, II, and III meningioma histopathology, age ≥18 years, and follow-up duration >6 months. Exclusion criteria included meningiomatosis, maximum diameter <2.0 cm, simultaneously discovered intracranial aneurysms, vascular malformations, intracranial hemorrhage, recent oral anticoagulant medications, and loss of follow-up.

A total of 333 cases were included, among which 66 patients underwent preoperative embolization. The decision of whether performing preoperative tumor embolization usually depends on presence of flowing void effects on magnetic resonance imaging (MRI) or rich vasculature on CTA. However, these were not the strict protocols, and the choices were usually made depending on surgeons’ experiences. All procedures were performed under the approval of the ethics committee of the Southwest Hospital Affiliated to Army Medical University (Ethics Approval No. KY2021150). Written consents were acquired for all surgical procedures. Informed patient consent for the data collection and analysis was waived by the ethical committee due to the retrospective nature of the study.

### Cohort Matching

Before cohort matching, categories of patient- and tumor-related variables for controlling covariates between two groups were collected *via* medical records or PAC system. Patient-related variables included sex and age at diagnosis. Tumor-related variables included the tumor location indicated as convexity, falcine, anterior skull base, and medial skull base; tumor encasement of large cerebral arteries encompassing the internal carotid artery (ICA) and the middle cerebral artery (MCA) assessed from computed tomography angiography (CTA); major sinus invasion by tumor verified both from magnetic resonance venography (MRV) and surgery records; and maximum diameter assessed by preoperative MRI or CT.

Patients were divided into embolization and nonembolization cohorts, depending on whether preoperative tumor embolization was performed. To perform 1:1 ratio cohort matching, we implemented the propensity score matching algorithm with MatchIt Package (Version 4.3.2) (13) in R (Version 4.1.2). Parameters were set as follows: tumor location, tumor encasement of ICA/MCA, and sinus invasion by tumor were exactly matched between cohorts; tumor maximum diameter, patients’ age and sex were matched using *nearest-neighbor matching* method by default; and distance measurements were set to *glm* by default for propensity score matching. Quality of matches was assessed by *p*-value, eCDF statistics, jitter plots, eQQ plots, and Love plots.

### Clinical and Neuroimaging Assessment

Detailed neuroimaging, neuropathological, surgical, complication, and functional outcome data were acquired after successful cohort matching. Neuroimaging data were independently reviewed by two experienced doctors. Gross total resection (GTR) was verified as complete resection of the enhancing tumor mass on contrast-enhanced T1 MRI.

Surgical data included gross total resection data, surgery duration, estimated blood loss (EBL), volume of autologous blood transfusion and allogeneic blood transfusion, and preoperative and postoperative hemoglobin (HGB). Patients with postoperative brain herniation due to large hematoma, severe brain edema, or large hemispheric infarction, underwent secondary surgery to reduce the intracranial pressure by means of decompressive craniectomy and/or hematoma evacuation. Other surgery-related complications included death, hemiparesis, cranial nerve palsy, visual defect, decreased hearing, aphasia, mental disorder, intracranial infection, seizure, cerebrospinal fluid leakage, and respiratory failure.

Functional assessments were carried out with Modified Rankin Scale (mRS) both before and during follow-up. mRS decline was defined as follow-up mRS score increase by at least 1 grade when compared with preoperative mRS.

### Statistical Analysis

Continuous variables conforming to normal distribution were presented as *mean* ± *standard deviation* (*SD*), while continuous variables, that were not conforming to normal distribution, were presented as *median* (*interquartile spacing*). Student’s t-test was used for intergroup comparison when normal distribution and equal variance examination were met. In the contrary, Wilcoxon’s rank sum test was used for the comparison. Categorical data are presented as *frequency* (*percentage*). For comparison of categorical data between groups, Wilcoxon’s rank sum test or Chi-square test or Fisher’s exact test were performed. A *p* < 0.05 was considered to be statistically significant, and two-sided statistical tests were performed. All statistical analysis was performed in R (Version 4.1.2).

## Results

### Cohort Matching

By retrieving the electronic medical record system, a total of 452 patients underwent meningioma resection surgery at our institution, of which 333 patients meet our selection and exclusion criteria and included in the cohort-matching process. Preoperative embolization was performed only in 66 (19.82%) patients but not in the other 267 (80.18%). To reduce the confound effects of covariates, we performed 1:1 ratio propensity score matching. Sixty-six patients from the nonembolization group (*n* = 267) were matched to the preoperative embolization patient’s cohort and enrolled in the nonembolization group, with algorithm parameters described in the **Materials and Methods** section.

Between the embolization and nonembolization groups, the exact matching criteria about tumor location yielded successful matching (*p* = 1.00) compared with *p* = 0.50 before matching. Other exact controlling variables such as ICA/MCA encasement (*p* = 1.00 vs. *p* = 0.11) and sinus invasion (*p* = 1.00 vs. *p* = 0.44) also achieved successful matching. Categorical variable, sex, also achieved perfect matching, with *p* = 1.00 after matching compared with *p* = 0.33 without matching. For continuous variable max tumor diameter, propensity score matching increased the *p*-value of the comparison between two cohorts from *p* < 0.0001 to *p* = 0.69. Another continuous variable age at diagnosis, the matching method increased the *p*-value of the comparison between two cohorts from *p* = 0.77 to *p* = 0.90. *p*-value, eCDF statistics, jitter plot, eQQ plot, and Love plot also indicate satisfying matches between two cohorts (**Supplementary Table S1** and **Supplementary Figures S1**–**S3**).

### Patient Characteristics

The average ages at diagnosis of the embolization and nonembolization group were 56.64 ± 11.39 and 56.39 ± 11.16 years, respectively (*p* = 0.90). There were both 44 (66.67%) female cases in two cohorts (*p* = 1.00). The distribution of pathological grade in the embolization cohort was 51 (77.27%), 13 (19.70%), and 2 (3.03%) for WHO grades I, II, and III, while in the nonembolization group, the distribution was 58 (87.88%), 8 (12.12%), and 0 (0), respectively (*p* = 0.16). The maximum diameter of the tumor was 54.59 ± 15.84 mm in the embolization cohort versus 53.50 ± 15.07 mm in the nonembolization group (*p* = 0.69). The distribution of tumor laterality (left/right/midline) in the embolization cohort was 36 (54.55%), 28 (42.42%), and 2 (3.03%) and 34 (51.52%), 26 (39.39%), and 6 (9.09%) in the nonembolization cohort (*p* = 0.45). Tumors located along the convexity, falcine, anterior skull base, and middle skull base were 18 (27.27%), 24 (36.36%), 20 (30.30%), and 4 (6.06%) in both cohorts (*p* = 1.00). Approximately 10 (15.15%) cases were found to have ICA/MCA encasement in both groups. Sinus invasion occurred in 4 (10.60%) in both the embolization group and the nonembolization group (**Table 1**).

**Table 1:** Baseline clinical characteristics.

Characteristics	Embolization group (*n* = 66)	Nonembolization group (*n* = 66)	*p*
Age at diagnosis (years; mean ± SD)	56.64 ± 11.39	56.39 ± 11.16	0.90
Sex (female; %)	44 (66.67)	44 (66.67)	1.00
WHO grade			
I (%)	51 (77.27)	58 (87.88)	0.16
II (%)	13 (19.70)	8 (12.12)	
III (%)	2 (3.03)	0 (0)	
Maximal diameter (mm; mean ± SD)	54.59 ± 15.84	53.50 ± 15.07	0.69
Laterality			
Left (%)	36 (54.55)	34 (51.52)	0.45
Right (%)	28 (42.42)	26 (39.39)	
Midline (%)	2 (3.03)	6 (9.09)	
Location			
Falcine (%)	24 (36.36)	24 (36.36)	1.00
Anterior skull base (%)	20 (30.30)	20 (30.30)	
Convexity (%)	18 (27.27)	18 (27.27)	
Middle skull base (%)	4 (6.06)	4 (6.06)	
ICA/MCA encasement (%)	10 (15.15)	10 (15.15)	1.00
Sinus invasion (%)	4 (10.60)	4 (10.60)	1.00

### Surgery-Related Outcomes

Operative time in the embolization group was 302.50 (136) min and 300.00 (72) min in the nonembolization group (*p* = 0.48). There was no significant difference in gross total resection rate between the two groups (*p* = 0.68). The estimated blood loss was 600.00 (400.00) ml in the embolization group versus 500.00 (500.00) ml in the nonembolization group (*p* = 0.31). There was also no significant difference in the decrease of perioperative HGB level between the two groups, which is calculated as the preoperative HGB level minus the postoperative HGB level (*p* = 0.68). Thirty-five patients (53.03%) received blood transfusion during surgery in the embolization group compared with 28 (42.42%) patients in the nonembolization group (*p* = 0.35). There was no significant difference in the volume of blood transfusion in the two groups (*p* = 0.63) (**Table 2**).

**Table 2:** Comparisons of surgical outcomes between the embolization and nonembolization group.

Surgical outcomes	Embolization group (*n* = 66)	Nonembolization group (*n* = 66)	*p*
Surgical time [minutes; median (IQR)]	302.50 (136.00)	300.00 (72)	0.48
GTR (%)	49 (74.24)	51 (77.27)	0.68
EBL (ml; mean ± SD)	600.00 (400.00)	500.00 (500.00)	0.31
Decreasement of HGB (g/L; mean ± SD)	30.81 ± 15.82	26.59 ± 12.90	0.09
Blood transfusion (%)	35 (53.03)	28 (42.42)	0.35
Blood transfusion volume [ml; median (IQR)]	650.00 (657.50) (*n* = 35)	535.00 (875.00) (*n* = 28)	0.63

### Postoperative Complications

Postoperative complications were retrieved from both inpatient and postoperative outpatient follow-up medical records. Patients with postoperative brain herniation due to large hematoma, severe brain edema, and large hemispheric infarction underwent secondary emergency surgery to remove the blood clot and/or decompressive craniectomy. Two (3.03%) patients in the embolization group and 3 (4.54%) in the nonembolization group developed large intracranial hematoma postsurgery (*p* = 0.63). Two (3.03%) patients in the embolization group and 4 (6.06%) in the nonembolization group experienced postoperative large hemispheric infarction, brain edema, and chronic hemiplegia (*p* = 0.68). These patients were treated with decompressive craniectomy. One patient in the non-embolization group suffered from brain herniation due to postoperative hematoma and severe brain edema. Though emergency decompressive craniectomy and hematoma evacuation were performed, the patient unfortunately passed away.

Neurological deficits include hemiplegia, hemiparesis, CN VII palsy, visual defect, decreased hearing, aphasia, mental disorder, infection, and seizure (**Table 3**). Hemiparesis is the major postoperative neurological deficits in our observation, which occurred in 7 (10.61%) patients in the embolization group and 12 (18.18%) in the nonembolization group (*p* = 0.22).

**Table 3:** Comparisons of postoperative complications between the embolization and nonembolization group.

Complications	Embolization group (*n* = 66)	Nonembolization group (*n* = 66)	*p*
Brain herniation			
Postop hematoma (%)	2 (3.03)	3 (4.54)	1.00
Postop infarction (%)	2 (3.03)	4 (6.06)	0.68
Postop edema (%)	2 (3.03)	4 (6.06)	0.68
Hemiplegia (%)	2 (3.03)	4 (6.06)	0.68
Hemiparesis (%)	7 (10.61)	12 (18.18)	0.22
CN VII palsy (%)	1 (1.52)	1 (1.52)	1.00
Visual defect (%)	0 (0)	3 (4.54)	0.24
Decreased hearing (%)	0 (0)	1 (1.52)	1.00
Aphasia (%)	0 (0)	4 (6.06)	0.11
Mental disorder (%)	1 (1.52)	1 (1.52)	1.00
Infection (%)	0 (0)	1 (1.52)	1.00
Seizure (%)	2 (3.03)	1 (1.52)	1.00
CSF leakage (%)	0 (0)	1 (1.52)	1.00
Respiratory failure (%)	1 (1.52)	4 (6.06)	0.37
Mortality (%)	0 (0)	1 (1.52)	1.00
Patients with postoperative complications (%)	14 (21.21)	26 (39.39)	0.02

We observed a statistically significant lower rate of patients with at least one postoperative complication in the embolization group (21.21% vs. 39.39%, *p* = 0.02). *Post-hoc* analysis showed significantly higher level of estimated blood loss in the group of patients suffering from surgical complications (600.00 (525.00) vs. 500.00 (500.00) ml, *p* = 0.037).

### Functional Outcomes

The follow-up duration in our study ranged from 6.2 to 37.5 months. The last postoperative follow-up mRS score showed no significant difference between these two groups (*p* = 0.167). Because higher complication rate occurred in the nonembolization group, we wondered whether relevant complications would affect patients’ daily living independence. To compare preoperative mRS scores, we utilized decline of mRS score to assess worsening of patients’ functional independence after surgery. We observed a significantly higher rate of patients with mRS decline in the nonembolization group (31.81%) compared with the embolization group (15.15%) (*p* = 0.04) (**Table 4**).

**Table 4:** Comparisons of postoperative mRS score between the embolization and nonembolization group.

Last follow-up mRS score	Embolization group (*n* = 66)	Nonembolization group (*n* = 66)	*p*
0 (%)	43 (65.15)	36 (54.55)	0.167
1 (%)	11 (16.67)	9 (13.63)	
2 (%)	6 (9.09)	13 (19.70)	
3 (%)	2 (3.03)	4 (6.06)	
4 (%)	1 (1.52)	3 (4.55)	
5 (%)	1 (1.52)	1 (1.52)	
6 (%)	1 (1.52)	0	
mRS change			
Without mRS decline (%)	56 (84.85)	45 (68.18)	0.04
mRS decline (%)	10 (15.15)	21 (31.82)	

## Discussion

We presented a matched cohort study to compare the postoperative complications and long-tern functional outcomes in patients who underwent with/without preoperative embolization. Based on matching potential confounds between groups, such as patients’ age, sex, tumor location, tumor maximum diameter, sinus invasion, and MCA/ICA encasement, our results showed significant lower rates of postoperative complications and reduced mRS decline of patients with preoperative embolization. We did not find any significant differences between groups with respect to surgical outcomes, such as estimated blood loss, operation duration, and blood transfusion.

Surgical resection of large and highly vascularized meningioma is challenging due to life-threatening blood loss and related surgical risks. Preoperative endovascular devascularization sounds reasonable to reduce subsequent intraoperative blood loss. Though increasing clinical studies are being conducted since this technique was established almost four decades ago (2), discrepancies exist on whether patients would benefit from the manipulation (14, 15). Some groups reported reduced intraoperative blood loss (6, 8), softening of the tumor mass to facilitate the operation (5), and shortening of the operation duration (6, 9). However, other data suggest embolization associated with higher rates of neurological adverse events after surgery (16) and added risk for morbidity and mortality (14). Recent meta-analysis also reported controversial conclusion on whether preoperative embolization would be beneficial in terms of reducing the estimated blood loss and surgical time (10, 11).

It is noteworthy that most previous studies were conducted in a manner to enroll consecutive meningioma patients in their institute, without controlling possible patient- and tumor-related confounds (10, 12). It may result in heterogeneity between groups and limits the interpretation of their results. From our own experiences and previous literatures, factors such as large tumor size, unfavorable location, artery encasement, and sinus invasion may complicate the operation and produce potential risks (9, 17–19). Raper et al. analyze a total cohort of 470 meningioma patients and did not find any significant differences on surgery time and complications between embolization and nonembolization groups (7). Blood loss is significantly lower in the nonembolization group due to variances in baseline patient and tumor characteristics. As shown in their baseline characteristics, tumor location and maximum tumor diameter differ significantly between groups.

Przybylowski et al. firstly introduced retrospective cohort matching to control critical confounds between embolization and nonembolization groups to yield more convincing interpretation of comparisons (12). Their results indicated that preoperative embolization did not alter the surgical outcomes of patients but could lead to a greater chance of improving functional outcomes. However, their study only included WHO grade I intracranial meningiomas. In our study, WHO grades II and III meningiomas account for 17.4% of the total number of cases we investigated whereas other groups reported 10%–15% of all meningiomas (9, 20). More importantly, advanced WHO grades II and III meningiomas are associated with more aggressive behavior (21) which may complicate the surgical operation and produce potential risks. Thus, it is reasonable to include the more aggressive and advanced grade meningiomas in the current study.

Preoperative embolization was carried out in a minority of patients at our institution. With the large total consecutive meningioma surgery cases, we were able to perform a successful 1:1 ratio matching between the embolization and nonembolization groups, which met the matching criteria and minimized the influence of patient- and tumor-related confounds. Specifically, the maximum tumor diameter, which differs significantly between groups in the original unmatched dataset (*p* < 0.0001), reached a statistical intergroup balance after matching (*p* = 0.69). Such bias also exists in other studies (7, 12), which may indicate surgeons’ preferences to embolize potential risky meningiomas.

The rationales of preoperative embolization include the reduction of intraoperative blood loss and softening of the tumor mass to ease surgical operation and reduce surgery duration. Intriguingly, we did not find a significant improvement on the surgical outcomes of embolization, including estimated blood loss, surgical time, and volume of blood transfusion. The results are distinct from earlier studies (6, 9) but in line with Przybylowski’s findings (12). As discussed above, surgeons prefer embolization in patients with meningiomas that are highly vascularized and large, which may increase the chance to find differences on surgical outcomes. Another important issue is the time interval between endovascular embolization and cranial surgery. The greatest tumor softening may occur 7–9 days after embolization (5). At our institution, meningioma resection surgery is arranged within 24 h after embolization. This schedule takes into consideration tumor ischemia, necrosis (22), and edema which could contribute to elevated intracranial pressure postembolization. Within such short period, tumors may not reach the ideal softening point and thus limits the improvement of surgical outcomes, especially blood loss and surgical time.

**Figure 1:**
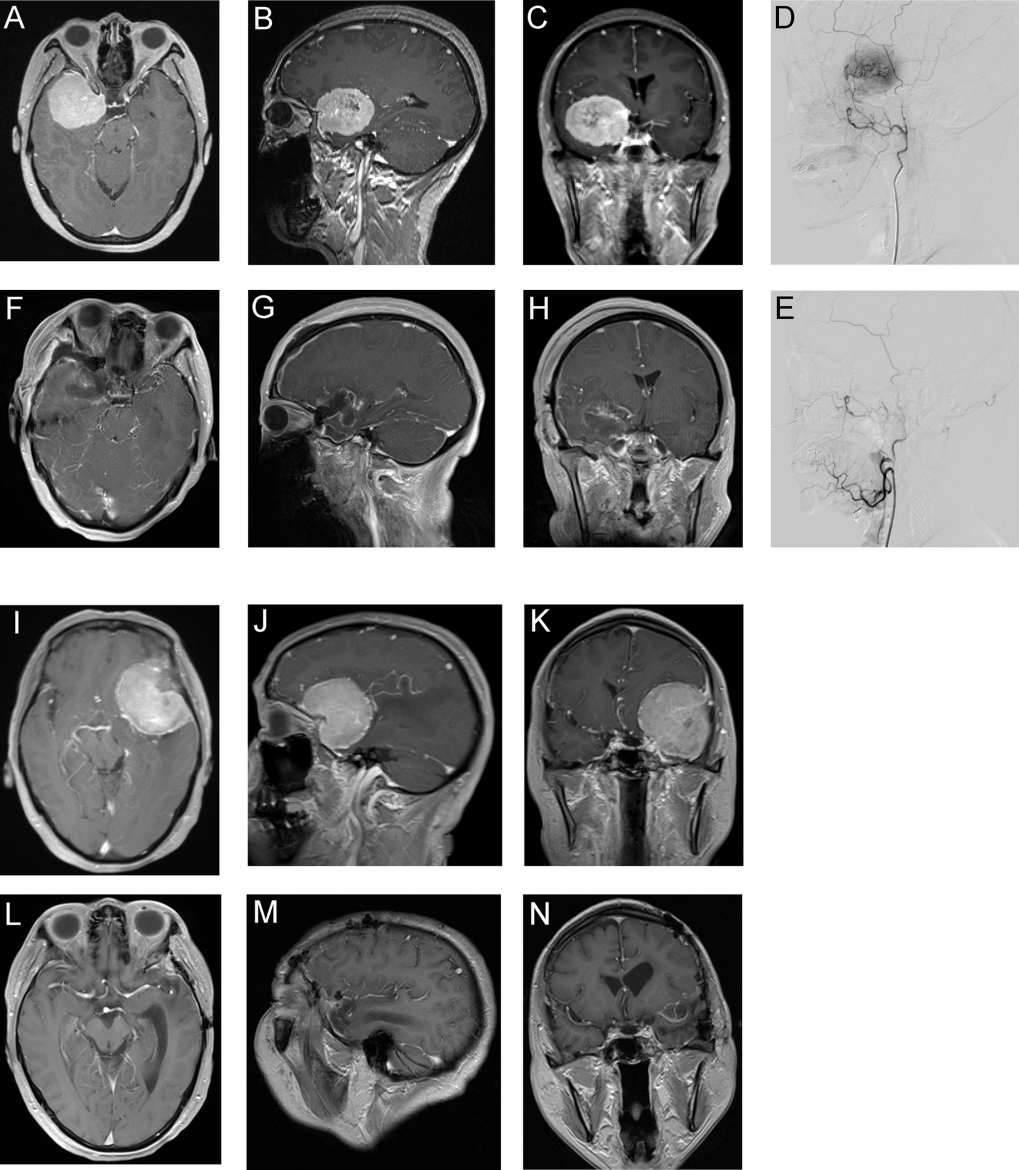
Two representative meningioma cases underwent preoperative embolization **(A–H)** or direct surgery **(I–N)**. Patient 1, 53-year-old woman. **(A–C)** Preoperative Gd-enhanced MRI showed sphenoid wing meningioma. **(D)** Lateral view of pre-embolization angiography showed hypervascular tumor feeding by branches originated from the middle meningeal artery. **(E)** Lateral view of postembolization angiography showed occlusion of the feeding vessel. The estimated blood loss of patient 1 was 500 ml and did not receive blood transfusion. **(F–H)** Postoperative Gd-enhanced MRI of patient 1. Patient 1 discharged routinely without surgical complication and last follow-up showed mRS improved by 1 grade. **(I–K)** Preoperative Gd-enhanced MRI of patient 2, a 55-year-old woman with sphenoid wing meningioma. The estimated blood loss of patient 2 was 1,800 ml and total volume of blood transfusion was 1,680 ml. **(L–N)** Postoperative Gd-enhanced MRI of patient 2. The patient discharged with right-side hemiparesis and last follow-up mRS declined by 2 grades.

Our data showed that preoperative embolization could significantly reduce the rate of surgical complication and the possibilities of mRS decline, which were distinct from others (7, 12). Sensory and motor function deficits were the majority of postsurgical complications and contribute to degrees of daily life disabilities, as shown in **Tables 3** and **4**. However, we did not find statistically significant differences between groups regarding surgical outcomes as discussed above. We speculate several factors may contribute to these findings. To establish a clear surgical view, surgeons may use aggressive surgical maneuvers such as retraction and electrocauterization to deal with complex tumor feeding vasculatures when dissecting vascularity-rich meningiomas. It may increase the difficulty to protect the adjacent critical structures (9) and raise potential risks to damage the proximity eloquent cortex, cranial nerves, and deep feeding vessels, thus contributing to higher postoperative complications. Immune attacks were shown to play a critical role in surgical-induced brain injury (SBI), through inducing cell death and brain edema (23). Recent murine studies revealed the meninges host a rich reservoir of myeloid immune cells (24). The cells may traffic to the brain parenchyma under CNS injury and autoimmune conditions. Preoperative embolization may potentially block the infiltration of immune cells and reduce the surgery-induced immune injuries. The potential roles of meningeal immune cell repertoire in meningioma need to be further investigated in future studies. *Post-hoc* analysis showed that in cases who suffered from postsurgical complications, the estimated blood loss was significantly higher. As the two cases of sphenoid wing meningioma presented in **Figure 1**, the nonembolized one suffered from greater blood loss and postsurgical hemiparesis and long-term limb weakness. These data indicated that preoperative embolization reduces postoperative complications and long-term disability, possibly through improved operative feasibility and safety.

Though the results of the presented study indicate that preoperative embolization could reduce unfavorable outcomes of meningioma patients, requirements in identifying which population would benefit from embolization still exist. In our study, the decision largely depends on the surgeons’ personal experiences in consideration with the tumor characteristics obtained from preoperative MRI and/or CTA. To our current knowledge, there are no consensus or guidelines about which patient population are suitable for preoperative embolization. Iacobucci et al. and Raper et al. suggested that it is reasonable to consider extensive devascularization for large meningiomas, tumors located deep in the surgeon’s line of sight, tumors in proximity to eloquent cortical areas, and tumors without extensive calcification. Beyond these structural characteristics, functional MRI imaging might provide objective and quantitative assessments of vascularity of certain meningiomas and necessity of preoperative embolization. Adachi et al. utilized normalized cerebral blood flow values (nCBF) and CBF images obtained from dynamic susceptibility contrast perfusion-weighted imaging (DSC-PWI) to predict the necessity of preoperative embolization (25). Mayercik et al. provided a noninvasive approach using arterial spin labeling MRI (ASL-MRI) to identify hypervascular meningiomas (26). We believe these objective functional imaging modalities may provide a more precise risk stratification of meningioma surgery.

We recognized that our study has several limitations to be considered. Though we matched possible confounds, the retrospective nature limits the robustness of the results. The conclusions need to be validated in large multicenter controlled trials. Bias could also arise from the surgeons’ individual preferences and surgical skills. The time interval between embolization and resection in our study is much shorter than that was reported in literatures, which may reduce the possibility of understanding the benefits associated with good surgical outcomes. We did not perform subgroup analysis on the relationships between the extent of devascularization and the outcomes of patients. As assessment of the angiographic myocardial blush grade is sometimes subjective, we were unable to carry out advanced neuroimaging modalities such as DSC-PWI and ASL-MRI to predict the necessity of preoperative embolization.

## Conclusion

The single-center matched cohort retrospective study showed significant lower rates of surgical complications and long-term disabilities of meningioma patients with preoperative embolization. There was no significant difference in estimated blood loss, operation duration, and blood transfusion volume between the embolization and nonembolization groups. Future studies are needed to investigate which subset of meningioma patients would benefit from preoperative embolization by incorporating objective and quantitative imaging approaches.

## Data Availability Statement

The original contributions presented in the study are included in the article/**Supplementary Material**. Further inquiries can be directed to the corresponding authors.

## Ethics Statement

The studies involving human participants were reviewed and approved by the ethics committee of the Southwest Hospital Affiliated to Army Medical University (Ethics Approval No. KY2021150). Written informed consent for participation was not required for this study in accordance with the national legislation and the institutional requirements.

## Author Contributions

FL, XL, YY, and YC designed the experiments. YY, YL, XL, and ZJ collected the data. CZ and HG reviewed the radiological imaging. ZC, RH, and YC evaluated the dataset. YY and CZ performed the cohort matching. YL, YY, and ZJ performed statistical analyses. YY, YL, ZJ, XL, FL, and HF drafted the manuscript. All authors contributed to the article and approved the submitted version.

## Funding

This work was supported by grant from the National Natural Science Foundation of China (Grant number: 81802509).

## Conflict of Interest

The authors declare that the research was conducted in the absence of any commercial or financial relationships that could be construed as a potential conflict of interest.

## Publisher’s Note

All claims expressed in this article are solely those of the authors and do not necessarily represent those of their affiliated organizations, or those of the publisher, the editors and the reviewers. Any product that may be evaluated in this article, or claim that may be made by its manufacturer, is not guaranteed or endorsed by the publisher.

## Supplementary Material

Supplementary Figure 1 Jitter plots were shown to visualize the distribution of propensity scores of matched and unmatched cases from embolization and non-embolization group.

Supplementary Figure 2 eQQ plots were shown to visualize the balance on the covariates before and after cohort matching.

Supplementary Figure 3 Love plots were shown to visualize the balance via absolute standardized mean difference between groups before and after cohort matching, as distance were estimated using generalized linear model.

## References

[1] Ostrom QT Cioffi G Waite K Kruchko C Barnholtz-Sloan JS. CBTRUS Statistical Report: Primary Brain and Other Central Nervous System Tumors Diagnosed in the United States in 2014-2018. Neuro Oncol (2021) 23:iii1–iii105. doi: 10.1093/neuonc/noab200 34608945 PMC8491279

[2] Manelfe C Guiraud B David J Eymeri JC Tremoulet M Espagno J. Embolization by Catheterization of Intracranial Meningiomas. Rev Neurol (Paris) (1973) 128:339–51. 4794375

[3] Dean BL Flom RA Wallace RC Khayata MH Obuchowski NA Hodak JA. Efficacy of Endovascular Treatment of Meningiomas: Evaluation With Matched Samples. AJNR Am J Neuroradiol (1994) 15:1675–80. PMC8333711 7847212

[4] Chun JY Mcdermott MW Lamborn KR Wilson CB Higashida R. And Berger, MS. Delayed Surgical Resection Reduces Intraoperative Blood Loss for Embolized Meningiomas. Neurosurgery (2002) 50:1231–1235; discussion 1235-1237. doi: 10.1097/00006123-200206000-00010 12015840

[5] Kai Y Hamada J Morioka M Yano S Todaka T Ushio Y. Appropriate Interval Between Embolization and Surgery in Patients With Meningioma. AJNR Am J Neuroradiol (2002) 23:139–42. PMC7975496 11827886

[6] Fang QR He XY Li XF Zhang X Chen M Li H. Comparative Efficacy of Glubran and Polyvinyl-Alcohol Particles in the Embolization of Meningiomas. Int J Neurosci (2016) 126:1112–9. doi: 10.3109/00207454.2015.1134525 26707920

[7] Raper DM Starke RM Henderson F Jr. Ding D Simon S Evans AJ. Preoperative Embolization of Intracranial Meningiomas: Efficacy, Technical Considerations, and Complications. AJNR Am J Neuroradiol (2014) 35:1798–804. doi: 10.3174/ajnr.A3919 PMC7966288 24722303

[8] Ishihara H Ishihara S Niimi J Neki H Kakehi Y Uemiya N. The Safety and Efficacy of Preoperative Embolization of Meningioma With N-Butyl Cyanoacrylate. Interv Neuroradiol (2015) 21:624–30. doi: 10.1177/1591019915590537 PMC4757340 26116646

[9] Iacobucci M Danieli L Visconti E Maresca M Anile C Colosimo C. Preoperative Embolization of Meningiomas With Polyvinyl Alcohol Particles: The Benefits are Not Outweighed by Risks. Diagn Interv Imaging (2017) 98:307–14. doi: 10.1016/j.diii.2016.08.006 27671861

[10] Jumah F Aburmilah A Raju B Jaber S Adeeb N Narayan V. Does Preoperative Embolization Improve Outcomes of Meningioma Resection? A Systematic Review and Meta-Analysis. Neurosurg Rev (2021) 44:3151–63. doi: 10.1007/s10143-021-01519-z 33723970

[11] Chen L Li DH Lu YH Hao B Cao YQ. Preoperative Embolization Versus Direct Surgery of Meningiomas: A Meta-Analysis. World Neurosurg (2019) 128:62–8. doi: 10.1016/j.wneu.2019.02.223 30954743

[12] Przybylowski CJ Zhao X Baranoski JF Borba Moreira L Gandhi S Chapple KM. Preoperative Embolization Versus No Embolization for WHO Grade I Intracranial Meningioma: A Retrospective Matched Cohort Study. J Neurosurg (2020) 134:693–700. doi: 10.3171/2020.1.JNS19788 32217797

[13] Ho D Imai K King G Stuart EA. MatchIt: Nonparametric Preprocessing for Parametric Causal Inference. J Stat Softw (2011) 42:28. doi: 10.18637/jss.v042.i08

[14] Shah AH Patel N Raper DM Bregy A Ashour R Elhammady MS. The Role of Preoperative Embolization for Intracranial Meningiomas. J Neurosurg (2013) 119:364–72. doi: 10.3171/2013.3.JNS121328 23581584

[15] Singla A Deshaies EM Melnyk V Toshkezi G Swarnkar A Choi H. Controversies in the Role of Preoperative Embolization in Meningioma Management. Neurosurg Focus (2013) 35:E17. doi: 10.3171/2013.9.FOCUS13351 24289125

[16] Wirsching HG Richter JK Sahm F Morel C Krayenbuehl N Rushing EJ. Post-Operative Cardiovascular Complications and Time to Recurrence in Meningioma Patients Treated With Versus Without Pre-Operative Embolization: A Retrospective Cohort Study of 741 Patients. J Neurooncol (2018) 140:659–67. doi: 10.1007/s11060-018-2996-0 30196368

[17] Champagne PO Lemoine E Bojanowski MW. Surgical Management of Giant Sphenoid Wing Meningiomas Encasing Major Cerebral Arteries. Neurosurg Focus (2018) 44:E12. doi: 10.3171/2018.1.FOCUS17718 29606042

[18] Ilyas A Przybylowski C Chen CJ Ding D Foreman PM Buell TJ. Preoperative Embolization of Skull Base Meningiomas: A Systematic Review. J Clin Neurosci (2019) 59:259–64. doi: 10.1016/j.jocn.2018.06.022 30279120

[19] Meling TR Da Broi M Scheie D Helseth E. Meningiomas: Skull Base Versus Non-Skull Base. Neurosurg Rev (2019) 42:163–73. doi: 10.1007/s10143-018-0976-7 29627874

[20] Shah A Choudhri O Jung H Li G. Preoperative Endovascular Embolization of Meningiomas: Update on Therapeutic Options. Neurosurg Focus (2015) 38:E7. doi: 10.3171/2014.12.FOCUS14728 25727229

[21] Perry A Louis D Budka H von Deimling A Sahm F Rushing E. Meningioma. In: Louis D Hiroko O Wiestler O Cavenee W, editors. WHO Classification of Tumours of the Central Nervous System, 4th. Lyon: IARC (2016). p. 232–45.

[22] Nania A Granata F Vinci S Pitrone A Barresi V Morabito R. Necrosis Score, Surgical Time, and Transfused Blood Volume in Patients Treated With Preoperative Embolization of Intracranial Meningiomas. Analysis of a Single-Centre Experience and a Review of Literature. Clin Neuroradiol (2014) 24:29–36. doi: 10.1007/s00062-013-0215-0 23525407

[23] Travis ZD Sherchan P Hayes WK Zhang JH. Surgically-Induced Brain Injury: Where Are We Now? Chin Neurosurg J (2019) 5:29. doi: 10.1186/s41016-019-0181-8 32922928 PMC7398187

[24] Cugurra A Mamuladze T Rustenhoven J Dykstra T Beroshvili G Greenberg ZJ. Skull and Vertebral Bone Marrow are Myeloid Cell Reservoirs for the Meninges and CNS Parenchyma. Science (2021) 373:eabf7844. doi: 10.1126/science.abf7844 34083447 PMC8863069

[25] Adachi K Murayama K Hayakawa M Hasegawa M Muto J Nishiyama Y. Objective and Quantitative Evaluation of Angiographic Vascularity in Meningioma: Parameters of Dynamic Susceptibility Contrast-Perfusion-Weighted Imaging as Clinical Indicators of Preoperative Embolization. Neurosurg Rev (2021) 44:2629–38. doi: 10.1007/s10143-020-01431-y 33215366

[26] Mayercik V Ma M Holdsworth S Heit J Iv M. Arterial Spin-Labeling MRI Identifies Hypervascular Meningiomas. AJR Am J Roentgenol (2019) 213:1124–8. doi: 10.2214/AJR.18.21026 31361532

