# Supplementary figures and images for: Clinical Outcomes and Complications of Preoperative Embolization for Intracranial Giant Meningioma Tumorectomy: A Retrospective, Observational, Matched Cohort Study
Source: Front Oncol. 2022 Mar 8;12:852327. doi: 10.3389/fonc.2022.852327 (PMC8957910; doi:10.3389/fonc.2022.852327)

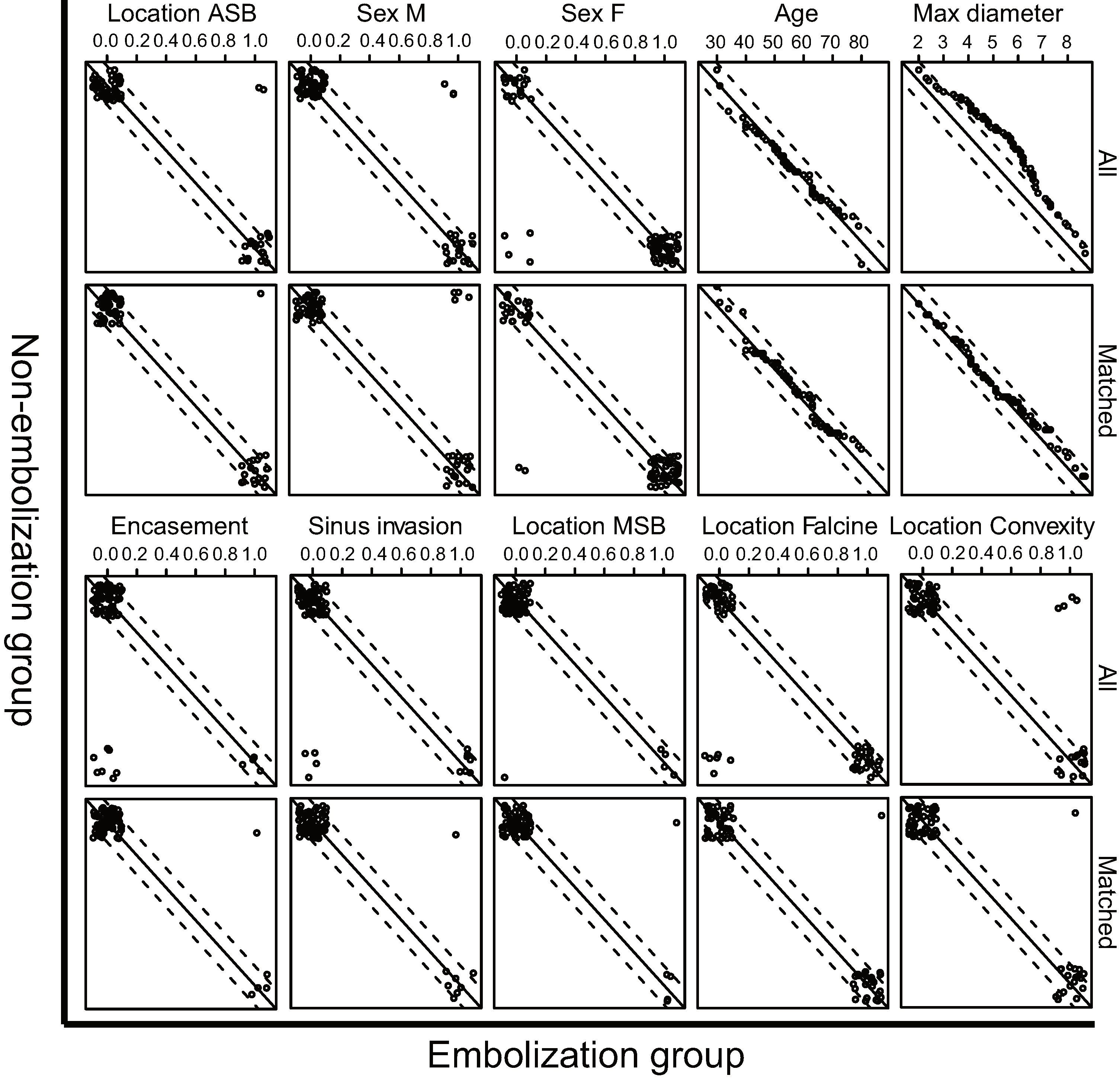

Supplement: Supplementary Figure 1 — Jitter plots were shown to visualize the distribution of propensity scores of matched and unmatched cases from embolization and non-embolization group. [file Image_1.tif]

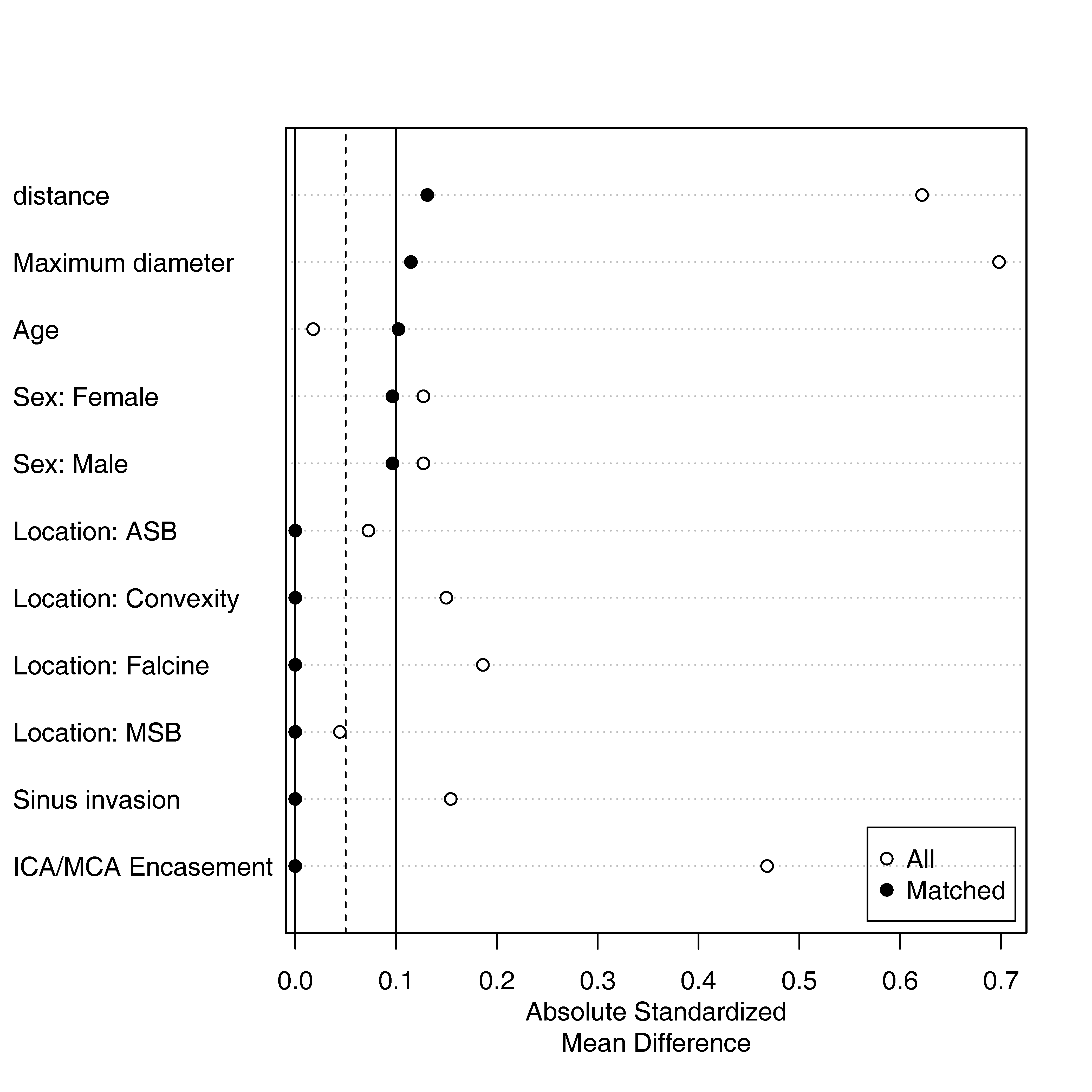

Supplement: Supplementary Figure 2 — eQQ plots were shown to visualize the balance on the covariates before and after cohort matching. [file Image_2.tif]

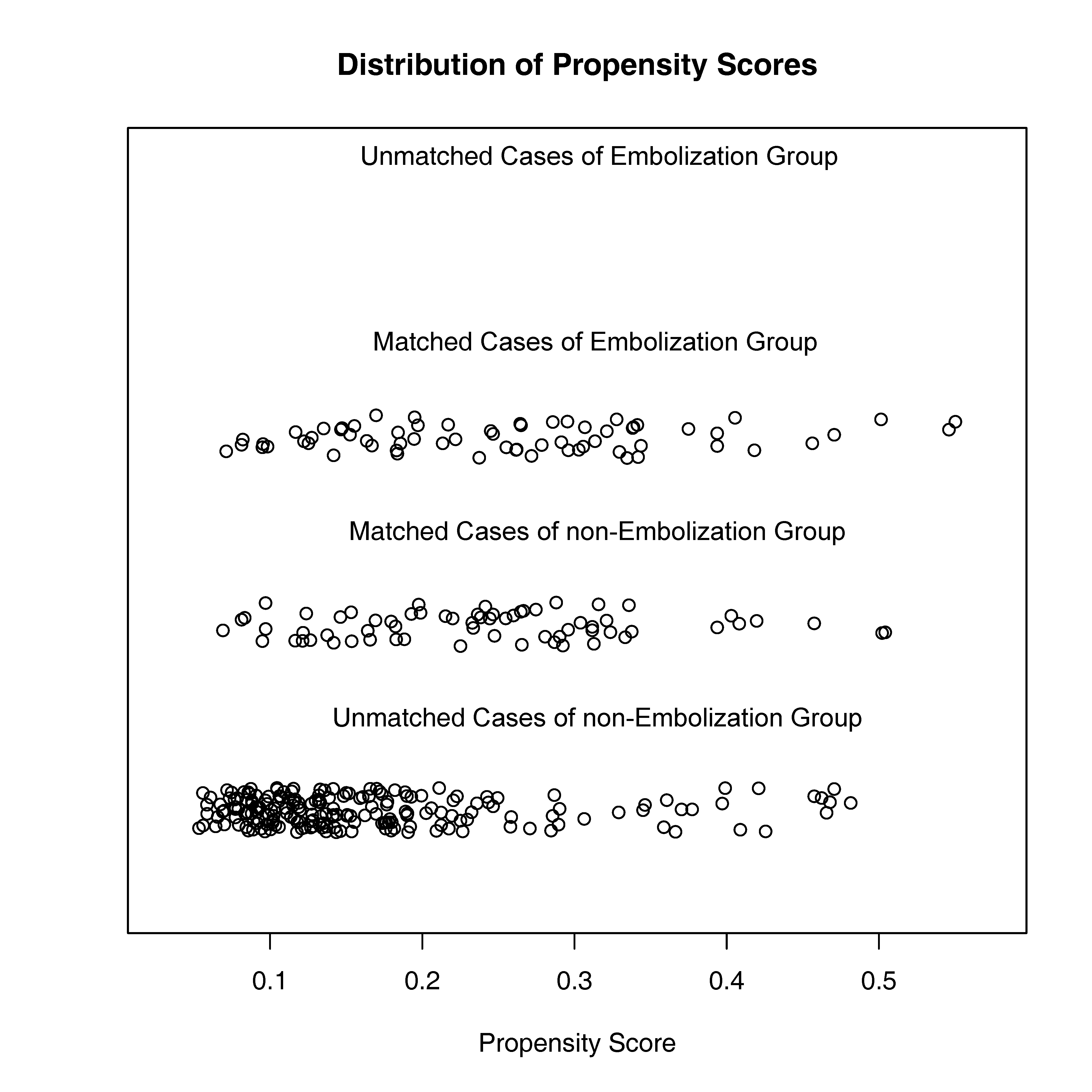

Supplement: Supplementary Figure 3 — Love plots were shown to visualize the balance via absolute standardized mean difference between groups before and after cohort matching, as distance were estimated using generalized linear model. [file Image_3.tif]
