# Supplementary material for: Clinical Outcomes and Complications of Preoperative Embolization for Intracranial Giant Meningioma Tumorectomy: A Retrospective, Observational, Matched Cohort Study
Source: Front Oncol. 2022 Mar 8;12:852327. doi: 10.3389/fonc.2022.852327 (PMC8957910; doi:10.3389/fonc.2022.852327)
Supplement: Supplementary file 4 [file Table_1.docx]

## Supplementary Table 1 eCDF and *p*-Value before and after cohort matching.

| Characteristics | eCDF  mean (max)  before matching | eCDF  mean (max)  after matching | *p*-Value before matching | *p*-Value after matching |
| --- | --- | --- | --- | --- |
| Age at diagnosis  (years; mean ± SD) | 0.0234 (0.0581) | 0.0233 (0.0758) | 0.77 | 0.90 |
| Sex (Female) | 0.0599 (0.0599) | 0 (0) | 0.33 | 1.00 |
| Sex (Male) | 0.0599 (0.0599) | 0 (0) |  |  |
| Maximal diameter  (mm; mean ± SD) | 0.9818 (0.1561) | 0.0183 (0.0909) | < 0.0001 | 0.69 |
| Location |  |  |  |  |
| Falcine (%) | 0.0895 (0.0895) | 0 (0) | 0.50 | 1.00 |
| Convexity (%) | 0.0667 (0.0667) | 0 (0) |  |  |
| ASB (%) | 0.0334 (0.0334) | 0 (0) |  |  |
| MSB (%) | 0.0106 (0.0106) | 0 (0) |  |  |
| ICA/MCA encasement (%) | 0.0705 (0.0705) | 0 (0) | 0.11 | 1.00 |
| Sinus invasion (%) | 0.0363 (0.0363) | 0 (0) | 0.44 | 1.00 |
